# Causes of Patient Nonattendance at Medical Appointments: Protocol for a Mixed Methods Study

**DOI:** 10.2196/46227

**Published:** 2023-11-03

**Authors:** Daria Schwalbe, Morten Sodemann, Maria Iachina, Bente Mertz Nørgård, Nina Høy Chodkiewicz, Jette Ammentorp

**Affiliations:** 1 Centre for Patient Communication (CFPK) Department of Clinical Research Odense University Hospital, University of Southern Denmark Odense Denmark; 2 Centre for Culture and the Mind (DNRF Centre of Excellence) Department of English, German and Romance Studies University of Copenhagen Copenhagen Denmark; 3 The Migrant Health Outpatient Clinic Odense University Hospital Odense Denmark; 4 Research Unit of Infectious Diseases Department of Clinical Studies University of Southern Denmark Odense Denmark; 5 Center for Clinical Epidemiology Odense University Hospital Odense Denmark; 6 Research Unit of Clinical Epidemiology Department of Clinical Research University of Southern Denmark Odense Denmark

**Keywords:** missed appointments, nonattendance, hospital appointments, Danish health care, prevention strategies, positive deviance, quality of treatment, mixed methods

## Abstract

**Background:**

Approximately one-third of patient appointments in Danish health care result in failures, leading to patient risk and sizable resource *waste*. Existing interventions to alleviate no-shows often target the patients. The underlying reason behind these interventions is a view that attendance or nonattendance is solely the patient’s problem. However, these interventions often prove to be ineffective and can perpetuate social biases and health inequalities, leaving behind patients who are more vulnerable or disadvantaged (in terms of social, economical, and linguistic factors, etc). A more holistic understanding of no-shows is needed to optimize processes, reduce waste, and support patients who are vulnerable.

**Objective:**

This study aims to gain a deep and more comprehensive understanding of the causes, mechanisms, and recurring patterns and elements contributing to nonattendance at Danish hospitals in the Region of Southern Denmark. It emphasizes the patient perspective and analyzes the relational and organizational processes surrounding no-shows in health care. In addition, the study aims to identify effective communicative strategies and organizational processes that can support the development and implementation of successful interventions.

**Methods:**

The study uses mixed quantitative-qualitative methods, encompassing 4 analytical projects focusing on nonattendance patterns, patient knowledge and behavior, the management of hospital appointments, and in situ communication. To address the complexity of no-shows in health care, the study incorporates various data sources. The quantitative data sources include the electronic patient records, Danish central registries, Danish National Patient Registry, and Register of Medicinal Product Statistics. Baseline characteristics of patients at different levels are compared using chi-square tests and Kruskal-Wallis tests. The qualitative studies involve observational data, individual semistructured interviews with patients and practitioners, and video recordings of patient consultations.

**Results:**

This paper presents the protocol of the study, which was funded by the Novo Nordisk Foundation in July 2022. Recruitment started in February 2023. It is anticipated that the quantitative data analysis will be completed by the end of September 2023, with the qualitative investigation starting in October 2023. The first study findings are anticipated to be available by the end of 2024.

**Conclusions:**

The existing studies of nonattendance in Danish health care are inadequate in addressing relational and organizational factors leading to hospital no-shows. Interventions have had limited effect, highlighting the Danish health care system’s failure to accommodate patients who are vulnerable. Effective interventions require a qualitative approach and robust ethnographic data to supplement the description and categorization of no-shows at hospitals. Obtaining comprehensive knowledge about the causes of missed patient appointments will yield practical benefits, enhancing the safety, coherence, and quality of treatment in health care.

**International Registered Report Identifier (IRRID):**

PRR1-10.2196/46227

## Introduction

### Background

Approximately one-third of all scheduled appointments at Danish hospitals do not succeed—that is, they are unattended by the patients [1]. An unattended or “missed appointment” is “when a patient does not show up for a scheduled appointment without sufficient notification, or any notification at all” [2,3]. At Odense University Hospital (OUH), for instance, every 20th patient does not show up for outpatient visits [1]. At Hospital Lillebælt, 12,424 cases of no-shows were registered during the first 7 months of 2018, which corresponds to approximately 4% of all appointments [4]. In the Capital Region, every fifth patient misses their hospital appointment [5]. No-shows are expensive owing to the large waste of resources that they entail. A single no-show costs health care between DKK 1500 and DKK 2000 (approximately US $227 to US $235). Bispebjerg Hospital spends DKK 40 million (approximately US $6 million) on nonattendances annually, whereas OUH spent as much as DKK 2.4 billion (US $380 million) on nonattendances in 2010 [6]. More importantly, no-shows increase the risk of patient errors and may seriously complicate patient treatment and continuity of care [7]. According to a Danish national report, “patients who do not show up for planned examinations, operations, etc., are a problem for the efficient use of resources in the healthcare system. It prevents other patients from arriving earlier, wastes staff time and prevents technology and operating theaters from being optimally utilised” [8].

The large number of no-shows for treatment in Danish hospitals has led to a lively debate among Danish politicians and in the daily press. So far, however, most papers that have addressed no-shows in outpatient and hospital appointments adopted an organizational perspective, ascribing no-shows to the faulty behavior of the patients (hence, referred to as “nonattendance”). It is the patient who “does not show up for hospital appointments," "has forgotten to cancel an appointment," or "is unable to show up for an appointment for other reasons” [5]. This is despite the fact that practices surrounding nonattendances vary significantly across various hospitals and sectors. At some clinics, it will be categorized as a missed appointment if a patient is at least 5 minutes late [2]. Such framing of the problem draws our attention toward individual behavior and no-shows as responsibilities of the patient and away from the structural and organizational shortcomings, which are responsibilities of the health care system. This perspective has been widely accepted among Danish policy makers, administrators, and health professionals, who generally view no-shows as a problem caused by patients. Some, for example, have suggested putting nonattending patients at the back of the queue for examination and treatment or giving them fines [9,10].

Most interventions to reduce nonattendance in Danish hospitals and outpatient clinics therefore target the patients: the introduction of web-based appointment systems, reminders via SMS text message, reminder letter, and so on. In Denmark and Great Britain, attempts have also been made to give the nonattendant patients fines. However, interventions such as fines are based on a lack of understanding of the health care system’s own role in missed appointments, and their effect is limited [11]. Other studies have claimed that up to 40% of nonattendances in health care are actually caused by errors on the hospital’s side [12,13]. It indicates that the many failures to attend hospital appointments may rather be “a symptom” of the shortage within the health care system itself to adequately accommodate patients who are more vulnerable. By “vulnerable patients” we mean those patients who have a low social capital or who belong to marginalized or socially excluded groups because of factors such as ethnicity, age (older people), language, low education level, low income, unemployment, housing conditions (eg, people experiencing homelessness), drug and alcohol addictions [14], people with complex illness (eg, type 2 diabetes) [15], and those individuals who do not have the capacity to take care of themselves (eg, children, people who are physically and mentally disabled, and people with mental illnesses) [14]. Vulnerability may also arise as a result of misunderstandings that lead to failure to fulfill the patient’s needs. In other words, it arises in the encounter between the physician and the patient, for example, as a result of misunderstanding or dissimilar points of departure or beliefs for the patient and the physician or health system, or forms of treatment [16].

Despite this, we found no documented interventions that target the health care causes for missed appointments. In the numerous attempts to reduce nonattendances at hospitals in Denmark, the patients’ perspective has also been lacking, and in general, we have very sparse knowledge about how relational and clinical factors affect patients’ no-shows. National, empirical studies of relational and organizational causes of nonattendances are practically nonexisting. Most no-shows in the Danish health care system are registered automatically but rarely with documentation of the cause. The causes of nonattendance in, for example, electronic patient records (EPRs), which are a platform that integrates electronic health records from hospitals [17], are not fully documented or not documented at all. The EPR only provides response options that deal with patient causes—not the administrative or clinical causes.

Previous studies that adopt the health care system perspective (with its fundamental understanding that patient are the ones who fail to attend hospital appointments) primarily focuses on (rather limited) sociodemographic descriptions of the patients who “do not show up” for hospital appointments (age, employment, socioeconomic status, ethnicity, etc). Furthermore, many studies have inadequate documentation or ambiguity regarding the reasons for these missed appointments. In addition, only a small number of studies have explored the patient perspective about this issue. Consequently, there is a lack of *a substantial body of research* that adequately explains the reasons behind no-shows. Our aim is to delve deep into the patient perspective and provide a more comprehensive understanding of no-shows at Danish hospitals.

### Determinants of Nonattendance (Literature Review)

#### Overview

**Figure 1:**
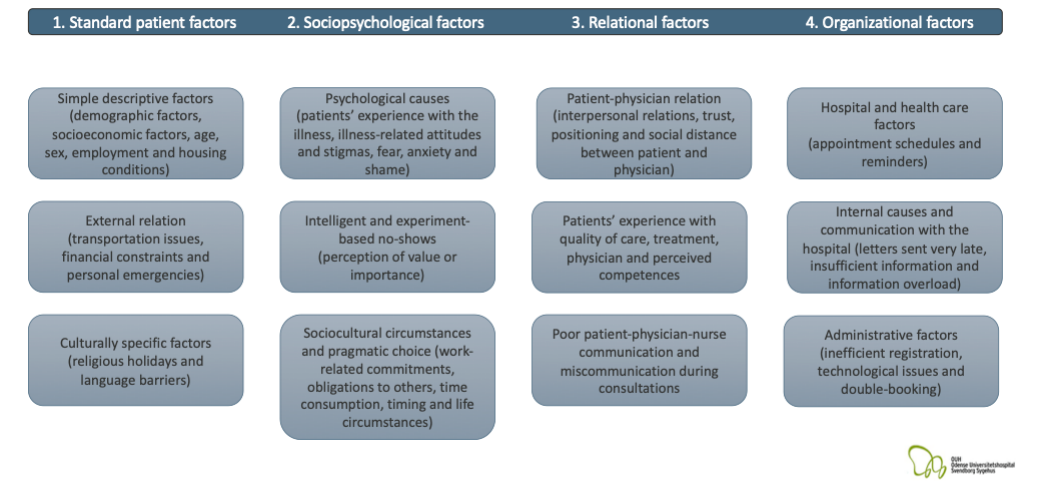
Determinants for nonattendance.

This project builds on previous studies of missed appointments or nonattendance and related interventions in hospitals and outpatient settings. Both Danish and international studies were included. In our review of literature, we have singled the following 4 key groups of factors and circumstances of “nonattendance” (refer to Figure 1).

#### Standard Patient Factors

A large number of studies of no-shows or nonattendances in health care—and particularly no-shows at Danish hospitals and outpatient clinics—focus merely on describing standard patient factors (such as age, sex, education, employment, etc), showing that there is a correspondence of nonattendance with sociodemographic factors such as age, ethnicity, sex, illness, insurance coverage, poor residence areas, socioeconomic status, timing of the appointment, travel time, transportation to and from hospital, and so on [18,19]. According to a Danish study, patients who fail to attend appointments are young people aged between 17 and 40 years, and they come from socially disadvantaged groups or from low-income housing areas, are unskilled, and have a high rate of unemployment [8]. The combination of long transport time and low income or low education are particularly intensifying when it comes to nonattendance [20]. Patients on social benefits, those with low income or short education, and those who have difficulty in finding transportation means (eg, lonely people) are also more likely to miss their appointments and have significantly more absences. Language (eg, patients who do not have Danish as their first language) and specific circumstances, such as religious holidays, may also be associated with nonattendance [8,19,21].

#### Sociopsychological Factors: Patient Knowledge, Risk Balancing, and Pragmatic Choice

Few qualitative studies of nonattendances similarly point toward themes such as balancing the costs and benefits of the individual attendance (intelligent compliance), lack of trust in the health care system, obligation to other people, stigmatization, anxiety, and patient’s attitudes toward the health care staff and toward illness as possible causes of no-shows [22,23]. Studies of nonattendances among patients with chronic disease [23-26], for example, show that there is a direct connection between nonattendance and the patient’s knowledge, attitudes, and behavior in relation to illness (eg, the patient’s feeling about whether the medicine is working, the patient is not satisfied with the treatment, or the patient does not trust the physician’s skills). Stigmatization (eg, obesity stigma) and shame can also act as barriers to comprehensive and effective treatment of type 2 diabetes and can be significant reasons for the patient’s no-show for the appointment [27]; health-related anxiety was a primary factor in decisions to discontinue long-term follow-up care among young survivors of cancer [28]. The patient’s willingness and unwillingness to meet up for hospital appointments may similarly depend on the patient’s relation with and obligations toward other people (that are framed by norms and beliefs within a particular society), patient’s previous experiences with and trust toward the health care system [29], perception about own position in relation to the physician’s position and skills [30], and diagnosis [31]. Patients who are vulnerable may further lack the resources and social support they need to establish and maintain their appointments [28,30]. A recent Danish report on social inequality in health care encounters also shows that there are considerable differences in patients’ skills and opportunities to search for health information themselves and absorb knowledge about health and illness, partly because patients with low socioeconomic status have low degree of social and cultural capital, which gives them few opportunities to seek knowledge via different sources, for example, internet, professional sources, and social networks [30]. Thus, in contrast to standard patient factors (age, education, housing, and ethnicity), this category is more about the interplay between the sociocultural and psychological factors (eg, moral values, obligations to others, fear, anxiety, and stigmas related to illness) and practical constraints that can influence peoples’ pragmatic choice and capacity to attend a hospital appointment.

#### Relational Factors, Quality of Patient Consultations, and Patient’s Relation With the Treating Clinician

Relational factors relate to social interactions and communication in patients’ encounters with health professionals. It encompasses elements such as patients and health professionals’ competencies, attitudes, and expectations toward the encounter and other communicative mechanisms that create social distancing and distrust between patients and health professionals [30]. The role of the quality of patient-physician conversations and the relationship that clinicians and the patient have before no-show has not been investigated in the Danish context. However, international studies have shown that the patient’s perceptions about the treating physician may play an important role in nonattendance [32,33]. Clinical psychologists’ patients had the lowest proportion of nonattendances (7.8%), followed by consultant psychiatrists (18.6%), specialist registrars (34%), and senior housekeepers (37.5%) [34]. Some referring physicians have significantly more nonattendances than others, just as patients who are treated by the youngest physicians are most likely not to show up for their appointments [35]. A large study from Australia showed that 50% of patient compliance is related to trust toward the treating physician [36], whereas other studies have shown that the failures to attend appointments were caused by insufficient communication from the physician, for example, when the physician did not explain sufficiently why the given appointment is important for the patient [33].

#### Organizational Factors and Errors in Hospital Communication

Organizational factors relate to the organization of the health care system and how the conditions in the system enable or prevent the accommodation of some patients [30]. International studies, for instance, reveal that minimum 35% to 40% of nonattendances at hospitals are caused by errors on the part of the hospital—errors in administration of patient appointments (letters not sent, wrong times, wrong bookings, wrong appointment places, changed agreements, very short notices, etc), insignificant or redundant hospital appointments, lack of availability, insulting behavior or speech, slow mail correspondence, and other administrative matters for which patients are not accountable [12,37,38]. A study of nonattendance at surgical control after trauma hospitalization showed, for example, that 37% of nonattendances were solely owing to the lack of communication about the appointment between the surgeon and nurse or between the medical team and the patient, which resulted in miscommunication [39] and not because the patients simply forgot the appointments, as other studies have claimed [40]. Another study claims that 41% of nonattendances are caused by errors on the part of the hospital [41].

The nonattendance rates can also differ significantly among different hospitals and among different units at the same hospital. The nonattendance rate in medical outpatient clinics in Denmark is 12% to 13% compared with 16% to 20% in England [41], and some specialties (eg, orthopedic surgery and type 2 diabetes) are systematically overrepresented among the high nonattendance rates, whereas others most often have low nonattendance rates (eg, in cancer treatment). Although it can be difficult to compare the nonattendance rates across units, both because the percentage can be estimated differently (per patient or per course of treatment and ≥1 nonattendances/course of treatment) and because there is a significant difference in the severity of illness (for instance, being a patient with cancer; being a patient with a chronic illness such as diabetes; or having a disease burden owing to, for example, multimorbidity) [26,42], we see that in chronical specialties, patients with low attendance compliance weigh heavily, whereas very few patients are represented in repeated rehospitalizations [41].

There is also evidence suggesting that comorbidity, an increased number of relationship problems, difficult consultations, and the lack of attendance affect the continuity of care [7,43]. Among the factors that prevent patients from attending, for example, asthma assessment consultations at hospitals are memory loss, reduced mobility owing to poor health, and frustration over the organization of the clinic that results in long waiting times and interruptions of treatment [44]. Attachment is also important for shaping and understanding the clinician-patient relationship and is essential for adherence to treatment and continuity of care [45], whereas social networks can be decisive when it comes to supporting patients who are vulnerable [46].

All of this supports our claim that nonattendance is a complexly determined behavior and is not something purely driven by the patient. To understand this complexity, we therefore need to look beyond purely descriptive patient factors and pay more attention to relational and organizational aspects of health care encounters that may result in no-shows. To further understand cognitive processes (decision-making) behind no-shows, we also need to investigate the relationship between the sociopsychological factors and pragmatic conditions and the patient’s conceptual representations (knowledge and values) that influence the generation of no-shows. Such knowledge may also help to develop prevention approaches and interventions that are appropriate and relevant—that is, target specific causes for no-shows within a particular context and setting.

### Interventions That Enhance Attendance

Interestingly, interventions targeting the specific causes for nonattendance at outpatient and hospital appointments work and can significantly reduce the number of nonattendances. We know of 5 well-documented, simple interventions that can reduce nonattendances at hospitals and ensure equality [41,47-49]. However, most interventions related to nonattendances are aimed at patients (they receive a reminder, can book an appointment themselves, or they receive a fine for not showing up) and are not related to the errors and deficiencies triggered by the health care system. A hand surgery clinic, for example, established an open outpatient clinic unit, where patients who missed to attend an appointment could themselves book a new appointment, thereby reducing nonattendance from 16% to 11% [41], whereas the pediatric ward in Viborg achieved analogous results by changing their guidelines and introducing a reminder via SMS text message [48]. A Cochrane review documents that SMS text message, e-mail, postage-paid reply envelopes, and self-booking outpatient clinics all significantly reduce nonattendances [50,51].

In Denmark, different hospital units are currently introducing web-based appointment systems to give patients more responsibility and better opportunity to make decisions about their appointments. Nevertheless, according to the staff, some patients experience barriers related to using web-based booking systems (low computer skills, ignorance of the web-based appointment, distrust of the internet, preference for verbal communication, etc) and may therefore be reluctant to use the system [52].

Having knowledge about the experiences and needs of the specific target group and in-depth insights into organizational processes and methods for creating relationships with and for the patient is, therefore, crucial not only for understanding why health care appointments fail but also for improving patient compliance and continuity of care and for supporting interventions that work well.

### Aims of the Study

The intention of this project is to provide a deep and more comprehensive understanding of the causes, mechanisms, and recurring patterns and elements contributing to nonattendance at hospitals and outpatient clinics in Denmark. The key objectives are (1) to describe the pattern of nonattendance and patients’ experiences thereof, (2) to identify managerial practices and interactional strategies that work or do not work well, and (3) to identify personal factors and administrative processes that can predict nonattendance and that can support appropriate implementation effort. Keeping this in mind, we ask the following research questions:

1. How do patients explain why they miss their hospital appointments?
2. What is the role of the quality of patient consultation and the physician-patient relationship before nonattendance for whether the patient attends their appointment?
3. How are no-shows handled by hospitals and medical professionals; what actions are taken (or not taken); and how does it affect the outcome, communication, time, and workload?
4. Which interventions do questions 1 to 3 offer possibilities for?

We, thus, put emphasis on some of the determinants presented previously and adding new ones, as we collect both quantitative and qualitative data. To pursue the research objectives, we propose a mixed methods design that combines research methods from health science, anthropology, cognitive science, humanistic interaction research, health care communication, and translational science.

## Methods

### Overview

Given that the goal of the project is to enhance an understanding of the problems and opportunities associated with missed hospital appointments, it is essential to grasp both the general patterns regarding the occurrence of patients’ nonattendance in Danish hospitals, which are available and hold significance from an epidemiological and biostatistical perspective. In addition, we must delve into the atypical instances and trends of no-shows, which come to light through the use of ethnographic approaches. To address these goals, the project uses a mixed quantitative-qualitative approach and is divided into three phases:

1. Quantitative data collection and analysis
2. Qualitative data collection and analysis, organized into 3 subprojects
3. Identifying prevention strategies

**Figure 2:**
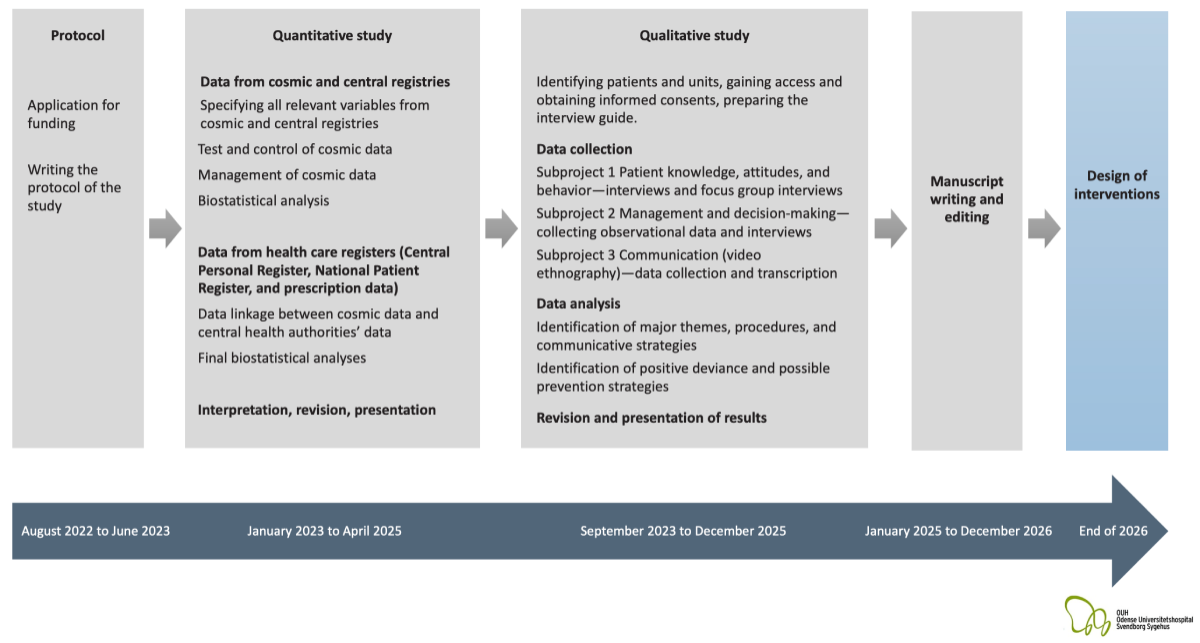
Overview of the project design and aims of the study.

In the first phase of the project period, the focus is on quantitative analysis. We will identify the recurring patterns of no-shows in the Region of Southern Denmark and the patients and hospital units eligible for the qualitative studies in the second, qualitative phase of the project. In the last phase, the achieved knowledge will be used to identify relevant prevention strategies and to design context-based, evidence-driven interventions that target specific causes of no-shows (refer to Figure 2 for an overview of the project design and aims of the study).

### Quantitative Data Collection and Analysis

#### Description of the Study Population

The study population of this project comprises all patients who are residents in the Region of Southern Denmark and who were referred with at least 1 contact to a hospital in the period between January 1, 2018, and December 31, 2022. Patients who do not turn up for an appointment owing to death or immigration will be excluded from the study population.

#### Data for Analysis

To describe the patient population and the pattern of nonattendance, we will retrieve a series of information including the patient’s age, sex, diagnostic codes for hospital contact, weight, height, smoking status, alcohol consumption, and information about planned and performed hospital examinations and treatments. In addition, we will investigate whether the patient’s other personal factors, such as cohabitation, number of children, working status, socioeconomic status, and medical history affect the risk of nonattendance. Nonattendance at hospital appointments is our primary interest. Information about nonattendance will be retrieved from the EPR system. Information about planned and performed examinations are to be applied as explanatory background variables. This information will be retrieved from the EPR system. Information from the Danish central registries for all patients in the study population will be used to retrieve information about the civil and vital status of the patients and about immigration and emigration from the civil registration system. Furthermore, we will retrieve information about all previous hospital contacts from the Danish National Patient Registry and about prescriptions from the Register of Medicinal Product Statistics. The central registers from Statistics Denmark are tools for retrieving information about the patients’ socioeconomic status and additional background variables.

#### Statistical Analyses

We will divide the patient population according to the nonappearance patterns on several levels: hospital level, department level, disease diagnosis level, and calendar time. The patient characteristics will be assessed according to these levels. We will use descriptive and more advanced analyses. *Chi-square tests* and *Kruskal-Wallis* *tests* will be used to compare the baseline characteristics of the patients across different levels. To estimate the odds ratio for nonattendance, we will conduct both univariate logistic regression analyses and multivariate logistic regression analyses, where we will adjust for various combinations of adjustment variables (including age, sex, socioeconomic status, type of underlying disease, and cohabitation).

### Qualitative Data Collection and Analysis, Organized Into 3 Subprojects

#### Description of the Study Population

The project uses the open approach, which means that units, medical professionals, and patients who are to be included in the qualitative study will be obtained through quantitative analysis. On the basis of the current picture, we will identify and investigate units (and practitioners) that are similar in clinical specialty but that lay on the opposite side of the spectrum regarding no-shows. We will further identify those patients whom we will follow throughout the course of their treatment and with whom we will conduct individual interviews to identify some important barriers and facilitators (even if we do not get hold of those patients who score highest on the no-show). To include ethnic minority individuals, an additional subgroup will be chosen among patients with an ethnic minority background who have been referred to the Migrant Health Outpatient Clinic at OUH during the same period. Ethnic minority individuals are often underrepresented in clinical studies [53] but are, as a rule, overrepresented among the nonattendances in survey studies—and often for reasons other than those of the majority population: the lack of knowledge about the purpose of control appointments, lack of reading skills, poor economy, transport barriers, and interpreting fees—that is, the fee that the patient has to pay for having an interpreter present during the consultation—are among the key reasons [54]. The inclusion of ethnic minority individuals is vital and will help to secure great validity and equality of the study. Only patients who agree to participate in the study will be included. Patients who die or move during the study period will be excluded from the study, unless we have obtained the necessary data.

#### Data for Analysis

To achieve more comprehensive data about missed appointments, we will investigate patient knowledge, attitudes and experiences related to missed appointments and identify to what extent organizational factors (tight day programs, excessive documentation, lack of time, etc) and relational dynamics (interpersonal relations between patient and physician or nurse, trust, empathy, attitudes, etc) contribute to the generation of no-shows and shape the pragmatic choice [55,56] and ways in which information is administrated and provided to the patient. It will provide a deep understanding of patient-related and disease-related factors, interactional dynamics, and organizational processes, which cause patients to miss their appointments at Danish hospitals and outpatient settings in the Region of Southern Denmark. It will also identify the role that the quality of patient consultations and the patient-physician relationship before nonattendance plays in the success or failure of attendances. This will help secure appropriate practices at hospitals and support the development of interventions based on situated data and the reasons for nonattendance. To meet the key goals, the qualitative part is divided into 3 independent but interrelated subprojects, focusing on patients’ knowledge, attitudes, and behavior (subproject 1); decision-making and management of hospital appointments (subproject 2); and interpersonal relations and communication (subproject 3).

#### Subproject 1

The *patient’s knowledge, attitudes, and behavior* subproject will examine the patient’s lived experiences and perceptions about the quality and course of treatment and their reasons, motives, and motivations for not showing up for hospital appointments by drawing on patient-reported outcome data about physical and mental health symptoms and quality of life and on in-depth semistructured qualitative interviews with selected patients [57,58]. To help secure patient perspective, the analysis of the project involves patient and relative representatives who are members of the research committee at the Centre for Clinical Epidemiology and of Center for Research Together with Patients and Relatives (*Center for Forskning Sammen med Patienter og Pårørende*; ForSa-P) [59] throughout the study to discuss and comment on the main aspects of the study. They will receive upgrades of the study progress on a regular basis and will be included in the evaluation of study results and relevance. To include ethnic minority patients and gain insight into their experiences with hospital agreements (and possible side effects), 3 separate focus group interviews with patients referred to the Migrant Health Outpatient Clinic at OUH during the same period as the main cohort will be conducted. We will focus on patients within 3 different languages and ethnic backgrounds, as these will overlap for most participants: Arabic, Somali, and Farsi or Turkish, as these immigrant groups are the largest within the Danish context. The number of participants for each group will be limited to 8. The focus groups will be facilitated by a Doctor of Philosophy student and conducted with the assistance of an interpreter and will focus on barriers related to and implications of no-shows experienced by those patients.

#### Subproject 2

The *decision-making and management of hospital agreements* subproject will explore how no-shows are managed by hospitals, hospital units, and medical professionals and the implications it has for the number of registered cases and for the frequency of nonattendance. A Danish study has claimed that the health care system’s capacity to accommodate some patients often relates to the limited resources within the health care system and may be explained by, for example, the time that is available for health professionals, their educational prerequisites for communicating with socially (and ethnically) diverse patients, and their knowledge about the available referential options and offers, which places demands on patients and any relatives to be able to navigate their course of treatment themselves [30]. The experience from a pilot study at OUH further shows that the management of hospital agreements often lies with the individual physicians and that certain structural factors (tight day programs, excessive documentation, and lack of time) can prevent the individual physicians from examining the individual cases further or contacting the absent patients. Moreover, physicians often use time to attend to other tasks. This raises the question about whether the time lost as a result of no-shows is actually wasted or whether opportunities arise instead to solve other tasks, such as, for example, documentation of patient progress or professional sparring.

To document how patient appointments and no-shows are managed locally (ie, when is the patient registered as “absent”; has the patient been contacted via phone and by whom; how many times should the patient be registered as “absent” before the case is closed; who closes the case; and what are the strategies, norms, and procedures; and what are the challenges?), this study will rely on systematic observations applied in cognitive ethnography [60,61]. Observation at the reception desk will be conducted by a research assistant or postdoc at 4 hospital units, identified in the first phase. They will be integrated into the clinic for a period of 1 month at each unit [58,60,62], producing a running record of mundane practices surrounding no-shows. To supplement observational data and secure their validity, the researcher will conduct individual semistructured ethnographic interviews [63] with medical secretaries and other medical professionals at each unit, to investigate their attitudes and how they handle no-shows (approximately 5-10 interviews at each site or unit, depending on the size of the unit). The study will be conducted in collaboration with the units, and the core informants will be identified by the researcher, in collaboration with the management team, in the course of fieldwork. We anticipate that the examination will enable us to identify gaps in the statistical data and provide insight into which actions are taken in connection with such situations. Administrator perspective can further provide key insights into organizational factors that influence no-shows. It can also help outline best practices, that is, define the managerial strategies that work best.

#### Subproject 3

The *interpersonal relations and communication* subproject will focus on patient consultations and examines how clinicians communicate with the patient in situated, real-time, real-life interaction. Communication (how we meet the patient; whether we manage to listen, communicate interest and care, and create security and trust; etc) is crucial to how patients experience their encounter with the health care system. Communication can both help to address nonattendance and cause it. It may also produce social inequality, for example, owing to patient’s ethnicity, poor hygiene, or poor language skills [64] or owing to social distance between the patient and the treating physician [31], which may then contribute to the patient’s no-shows. Subproject 3 seeks to examine the communicative strategies that may influence the patient-clinician relationship and trust (eg, time to listen, ability to read the patient or communication cues, ability to include and recognize the patient and the patient’s needs, interruptions, etc). In contrast to subproject 2, which aims to map managerial strategies and challenges within respective hospital units and therefore focuses on communication more broadly (“a focus on equal access to care for all”) than the patient-clinician relation, the objective of subproject 3 is to understand the extent to which the quality of patient consultation and the patient-clinician relationship (in particular, the one before no-show) plays a role in nonattendance. It therefore focuses more specifically on situated interactions and observations of live medical appointments between providers and patients, which can be examined through the application of video ethnography [65] and interpersonal dynamics that can contribute to a positive (or negative) dialectic relation between the clinician and the patient [66,67]. The video consultations will be conducted at 2 chosen hospital units, which will be identified based on quantitative analysis. The anticipated size of video sample is 60 to 100 patient consultations, depending on the number of participating units and medical professionals and the obtained consent. Combined with statistical data about the frequency of no-shows, these data may enlighten our understandings of the relational aspect of nonattendance. It may also help us to identify the strategies that work well.

#### Analytic Tools and Procedures

The 3 subprojects will share a common data pool consisting of ethnographic observational data (field notes), individual interviews with patients and practitioners, questionnaires regarding the patients’ satisfaction with consultations or patient-reported outcomes, and video recordings of patient consultations. In the analysis of the data, we rely on phenomenological-hermeneutic tradition [58] and the thematic analysis used in traditional ethnography [68,69] and medical anthropology [33,70,71] and on cognitive ethnography [72], which is a method for studying distributed cognition in organizational contexts. A distributed approach means that cognitive processes (eg, decision-making) are examined in relation to the sociocultural context in which they occur, as opposed to classical approaches focusing on mental processes in individuals [60]. A cognitive ethnographic approach provides an opportunity to scrutinize both the conditions and the implications that the cognitive processes have in a particular organization. It allows to scrutinize no-shows as a dynamic dialogical process, that is, something that unfolds in the interaction among the patient, the health care professionals, and the environment. Cognitive ethnography can also provide insights into existing challenges in coordination of, for example, patient agreements across hospital units and across sectors (sector transition) and can be particularly useful in addressing situated choice and decision-making regarding no-shows. We analyze experiences, perceptions, and behaviors through the lens of medical anthropology, identifying the key themes, recurring patterns, variations, and contradictions within and across the data [73]. We draw on cognitive ethnography [74-78] when analyzing how no-shows are managed in the clinical setting [75]. In our analysis of video data, we rely on multimodal interactional analysis [79] and on embodied participation framework [66,80]. To better understand the multifaceted aspects of interaction, we treat consultations as a multiactivity setting, where people engage simultaneously in >1 activity, involving “complex configurations of spatiality (built spaces that both afford and constrain), materiality (objects, artefacts, tools, representations, etc), and participation (varying levels of involvement, shifting orientations, and more)” [66]. Taken together, the ethnographic data will provide more comprehensive knowledge about the reasons, patterns, and processes behind no-shows.

### Identifying Prevention Strategies

This study intends to use a *positive deviance* approach to ensure the translation of the research to practice. The positive deviance method recognizes that within any society or organization, certain individuals or groups exhibit exceptional behavior and strategy that enable them to find better solutions to problems, despite having access to similar resources and facing similar or worse challenges. In other words, it focuses on those individuals (and, in our case, medical teams and hospital units) who demonstrate exceptional performance, despite facing the same constraints as others [81]. Positive deviance approach is increasingly used in health care organizations because it has the potential to solve many of the challenges faced by the organizations in the effort of enhancing quality [82]. A four-step process has been proposed to apply the positive deviation approach in health care: (1) positive deviations are identified using routinely collected data; (2) qualitative methods are used to generate hypotheses about how positive deviants succeed; (3) these hypotheses are tested in large, more representative samples; and finally, (4) the successful, positively deviant practices are widely disseminated [81,83].

Consistent with this approach, this part of the project aims to use the collected data to identify units, clinics, and individuals that exhibit exceptional performance and to identify practices and strategies that work well. Subsequently, a pilot study will be designed to test the effectiveness of the identified prevention strategies.

### Ethical Considerations

As of January 2023, the study was registered with the Danish Data Protection Agency—Datatilsynet (registry ID 22/54791) and with the Open Patient Data Explorative Network (registry ID OP_1800). Request for ethics approval was sent to the Regional Committees on Health Research Ethics for Southern Denmark [84]. The request has been given the case number 20222000–133. On October 26, 2022, the committee replied that, based on the available information, the committee has assessed that the project could not be considered as health science research with an obligation to report to the Health Research Ethics Committee System, as found in Committee Act Article paragraph 14, section 1. In the decision, emphasis has been placed on the fact that it appears to be a quality development project, where the trial-related procedures cannot be considered as an intervention within the meaning of the Committee Act. The project, therefore, falls outside the scope of the Committee Act’s definition of a reportable health science research project. Pursuant to the Committee Act Article paragraph 14, section 2, questionnaire surveys and register-based research projects must only be reported to the Research Ethics Committee System if the project includes human biological material. If a health research project is to fall within the framework for reporting to the committee system, the project must, as found in the guidelines from the National Committee on Health Research Ethics, both have a health scientific purpose and involve intervention.

## Results

Given that this paper is a study protocol, we do not yet have any results to report from the study. The study was funded by the Novo Nordisk Foundation and will be conducted for a period of 48 months (January 1, 2023, to December 31, 2026). The original title of the research application, funded by the Novo Nordisk Foundation, was “Non-attendant or not invited? A cross-disciplinary study about why healthcare appointments fail and how this can be prevented.” As of January 2023, the request for permission to use information from patient records has been directed to the Region of Southern Denmark [85], in accordance with the Health Act (Sundhedsloven) Article §46, section 2, and Article §47, section 1. Recruitment commenced in February 2023. It is anticipated that the quantitative data analysis will be completed by the end of September 2023, with the qualitative investigation beginning in October 2023 and the first study findings being available by the end of 2024. An overview of the project timeline and key activities is presented in Figure 2.

## Discussion

### Expected Outcomes

We are proposing an ambiguous study design, combining national registries and EPR with a longitudinal ethnographic study to explore a broad range of no-shows management, patient-specific experiences, and relational dynamics that cause no-shows. Rather than focusing on purely descriptive factors, this study rests on the idea that “nonattendance” is a complex problem—it implies societal and communicative or interactive challenges that are also tied to relational, sociocultural, and organizational factors. It further assumes that the long-lasting, narrow focus on the patient as the major problem and the key cause for nonattendance has prevented effective interventions to reduce the nonattendance waste, and it has produced more inequality with respect to patients, who are vulnerable; lack resources; or have previously been overlooked, excluded, or stigmatized.

The study aims to fill the major gap of knowledge about no-shows in the Danish health care context. It addresses structural, relational, and pragmatic dimensions of no-shows to provide a broad and more operational knowledge base for unsuccessful encounters between patients and health care professionals. In addition to providing a holistic picture of no-shows within the Danish context, this study also aims to identify the personal and organizational factors and administrative strategies that work well. We anticipate that such knowledge will yield significant practical advantages for health professionals and can be foundational in scaffolding better-informed development and implementation of interventions and prevention approaches.

### Limitations

Obtaining comprehensive and reliable data about no-shows can be challenging. The weakness of this study is that the registration of no-shows varies significantly across hospitals and even among units within the same hospital, and the quality of the data is not known. Hospital records may not always accurately capture the reasons for nonattendance, making it difficult to assess the underlying factors. Moreover, self-reported data rely on individual perceptions, attitudes, and beliefs. It may be biased and may not always provide an objective view of the reality [58,68]. To achieve more valid data, we combine methods such as ethnographic interviews [58], focus group interviews [86], systematic observations and video ethnography [65].

### Implications for Practice

The project will offer a methodological and empirical contribution to health science, humanities scholars, and medical professionals. It will contribute new insight by involving the patient perspective and analyzing the relational and organizational processes around no-show at hospitals and outpatient clinics in the Region of Southern Denmark. By incorporating various data sources and by linking national registers, EPR data, and patient satisfaction questionnaires with rich ethnographic (and video) data, it will create a comprehensive and multifaceted data pool. Taken together, these data will strengthen Danish health care by providing new and more comprehensive knowledge about the reasons, patterns, and processes behind no-shows. More comprehensive knowledge about problems and intervention possibilities (1) can partly be used to make predictions about nonattendances; (2) can help to identify interventions grounded in specific causes that trigger failures at hospital appointments; and (3) can identify patient perspectives and strategies that can lead to interventions in health care interaction with patients who are vulnerable, where the risk of misunderstandings and missed appointments is high. This will yield significant practical advantages at least in 4 different ways, with potential for creating more appropriate and effective interventions. It will allow us to do the following:

1. Identify specific areas within the health care system where interventions can be implemented to reduce resource wastage effectively
2. Address social inequalities related to missed appointments, ensuring that populations that are vulnerable receive equitable access to health care services
3. Enable active participation of the health care system by using patient-reported information and engaging in cocreation with patients to optimize resource allocation
4. Foster cross-sector collaboration, enhancing the overall coherence and quality of treatment

An approach to interventions that focus on “the good examples” can then be applied to others in the health care system and give clinicians and managements the opportunity to optimize organizational processes through targeted interventions. The project thus has the potential to reduce waste and clinical errors in health care, which will be beneficial for both the patients and the health care system. It also has the potential to cast light on how health care professionals can improve their skills for detecting potential nonattendants early in the process, for example, by articulating and responding to what matters to the individual patient.

In the future, the expanded model for determinants of no-shows could form the basis for algorithms created by machine learning, among other methods [87]. However, as artificial intelligence method of cogitation is mechanically distinct from human intelligence—computers lack emotion; thus, they cannot literally be courageous and their logic boards cannot process narrative [88]—jumping straight to machine learning is methodologically immature. It requires massive analytical resources, and it reproduces and intensifies the health care system’s own misconceptions and the existing administrative and professional discourses behind no-shows [89,90].

Finally, the project will also contribute to a new Research Portfolio for Human Factors and Patient Safety, established as part of a collaboration between the University of Southern Denmark and OUH. The portfolio is the first of its kind in Denmark, and it will enable researchers in close cooperation with clinicians to test and implement successful interventions and to further develop original ways of communicating and collaborating, to produce a more informed scholarly understanding of nonattendances in health care and the interaction of core biological, social, and cultural factors.

## Abbreviations

EPR: electronic patient record
ForSa-P: Center for Forskning Sammen med Patienter og Pårørende (Center for Research Together with Patients and Relatives)
OUH: Odense University Hospital

## Data Availability

According to Danish legislation, our approvals to use register data for this study do not allow us to distribute or make patient data available to other parties. Any interested researchers may apply for access to data through an application to the Research Service at Statistics Denmark and at the Danish Health Data Authority. Moreover, access to data from the Danish health data authorities requires approval from the Danish Data Protection Agency. The authors of this project do not have special access privileges to the data used in this study. The data that support the findings will be available from the Centre for Research in Patient Communication, University of Southern Denmark, but restrictions apply to the availability of these data, which were used under license for this study, and thus are not publicly available. Data available from the authors could be made available upon reasonable request and with permission of the University of Southern Denmark.
